# Effects of earthquake on perinatal outcomes: A Chilean register-based study

**DOI:** 10.1371/journal.pone.0191340

**Published:** 2018-02-23

**Authors:** Yasna K. Palmeiro-Silva, Pelusa Orellana, Pia Venegas, Lara Monteiro, Manuel Varas-Godoy, Errol Norwitz, Gregory Rice, Eduardo Osorio, Sebastián E. Illanes

**Affiliations:** 1 School of Nursing, Universidad de los Andes, Santiago, Chile; 2 School of Education, Universidad de los Andes, Santiago, Chile; 3 Faculty of Medicine, Universidad de los Andes, Santiago, Chile; 4 School of Medicine, Tufts University, Boston, Massachusetts, United States of America; 5 Centre for Clinical Research, Faculty of Medicine, University of Queensland, Brisbane, Australia; 6 Department of Obstetrics and Gynecology, Clinica Dávila, Santiago, Chile; University of Washington, UNITED STATES

## Abstract

**Background:**

Natural disasters increase the level population stress, including pregnant women, who can experience prenatal maternal stress, affecting the fetus and triggering perinatal complications, such as low birth weight, smaller head circumference, etc. However, little is known about effects of earthquake on perinatal outcomes.

**Objective:**

To evaluate the effect of earthquake occurred on February 27, 2010 and perinatal outcomes of Chilean pregnant women, and to examine these effects by timing of exposure during pregnancy and newborn gender.

**Methods:**

A register-based study was performed using data collected from women who had a vaginal delivery in a large private health center in Santiago, Chile, during 2009 and 2010. The study population was categorized according to exposure to earthquake and timing during gestation. Primary perinatal outcomes were gestational age at birth, birth weight, length and head circumference. Analyses adjusted for gender, gestational age at exposure, parity, maternal age and income.

**Results:**

A total of 1,966 eligible vaginal deliveries occurred during 2009 and 2,110 in 2010. Birth weight was not affected by the trimester of exposure; however, length, head circumference and gestational age at birth were significantly different according to trimester of exposure and gender of newborn. In multivariable analysis, newborns were shorter by 2 mm, 5 mm and 4.5 mm, if they were exposed during their first, second and third trimester, respectively. Furthermore, newborns had a smaller head circumference by 1.2 mm and 1.5 mm if they were exposed during first and second trimester of gestation.

**Conclusion:**

In this cohort, exposure to the February 2010 earthquake resulted in earlier delivery and reduced length and head circumference in the offspring. This association varied according to trimester of exposure and fetal gender. Health workers should include exposed to high levels of stress associated with natural disasters when assessing pregnancy risk factors.

## Introduction

The landmass on which Chile is located is highly seismically active. As a result, the country has experienced some of the world’s largest earthquakes. On February 27, 2010, a strong earthquake occurred 500 km south of Santiago with a magnitude of VIII on the Mercalli scale (8.8 Richter). This was the second strongest earthquake recorded in Chile [1] and the fifth largest ever recorded worldwide. It was followed twenty minutes later by a large tsunami, which flooded several coastal towns. Together, these natural disasters impacted the entire Chilean population, increasing hospital admissions for both acute medical and psychological conditions.

Pregnant women are especially vulnerable to natural disasters [2]. Moreover, exposure to such stressful events also affects the intrauterine environment and fetal development through a process known as in utero fetal programming. Prenatal maternal stress (PNMS), produced by exposure to natural disasters, has been associated with perinatal complications, such as low birth weight, low Apgar scores, smaller head circumference, higher rates of premature birth [3–12], suboptimal growth [13,14] and brain development [15]. Long-term complications have also been described, including higher rates of metabolic diseases [16,17], mental disorders, and obesity in childhood and adulthood [18,19]. It has been further suggested that the consequence of acute intrauterine stress may vary according to the timing of exposure and fetal gender [20,21].

Although exposure to excessive stress has long been known to affect the physical and mental health of individuals adversely, little is known about the effect of earthquakes and perinatal outcomes [3,6,9]. The aim of this study was to evaluate the effect of the earthquake, which occurred on February 27, 2010, on perinatal outcomes of Chilean pregnant women and to examine these effects by the timing of exposure during pregnancy and newborn gender.

## Materials and methods

### Study design

A register-based study was performed using demographic and routinely collected data on all pregnancies delivered at a single large private health center in Santiago (Metropolitan Region), Chile. This study received ethical approval from the Institutional Review Board at Clínica Dávila in Santiago. The study was conducted using retrospective and de-identified data; thus, no patient consent was required.

### Data

The database contained a total of 7,031 and 7,471 deliveries in 2009 and 2010, respectively. Subjects were excluded if they delivered outside of the study period (February 27 to December 11) in order to evaluate similar time-periods during pregnancy. Moreover, early preterm deliveries, C-sections, multiple pregnancies, and stillbirths also were excluded to avoid bias related to other complications that could trigger the process of delivery.

Women who delivered between February 27 and December 11, 2009, were classified as “non-exposed”; women who delivered during the same period in 2010 were classified as “exposed”. Women who delivered between September 6 and December 11 were categorized as being exposed (or non-exposed) during their first trimester, from May 31 to September 5 were regarded as being exposed (or not) during their second trimester, and between February 27 and May 30 were exposed (or not) during their third trimester.

All demographic and clinical information was abstracted and recorded by professional workers (medical doctors, midwives, and nurses). The data abstracted included: date of delivery, gestational age at delivery (in weeks), maternal age (in years), parity of mother, the location of residence, the gender of newborns, birth weight (in grams), length (in centimeters), head circumference (in centimeters), Apgar at 5 minutes, and small for gestational age (SGA) (defined as newborn birth weight <10th percentile for gestational age). Perinatal outcomes measured included: birth weight, length, ponderal index (weight/height^3^), head circumference, Apgar at 5 minutes, the proportion of SGA newborns, preterm delivery (birth between 34–37 weeks). Gender of newborns, location of residence, gestational age (either in weeks or days), parity and maternal age were considered as effect modifiers; location of residence was related to three categories of average annual income [22]: low income (500,000 to 1 million Chilean pesos), middle income (1 to 1.5 million Chilean pesos) and high income (more than 1.5 million Chilean pesos).

The current protocol was registered on www.protocols.io and the assigned DOI is dx.doi.org/10.17504/protocols.io.mguc3ww

### Statistical analysis

Normal distribution of continuous variables was assessed by Shapiro-Wilk tests. Quantitative non-normally-distributed variables are presented as median and 25^th^ - 75^th^ percentile. Normally-distributed variables are presented as the mean and standard deviation (SD). Qualitative variables are presented as percentages. Normally-distributed data were analyzed using two-tailed Student’s t-tests, and Wilcoxon-Mann-Whitney tests were used for non-normally distributed data for two groups comparisons. Meanwhile, ANOVA and Kruskal-Wallis tests were used for comparisons between the three groups, as appropriate. Chi^2^ tests were used to analyze proportions between the two groups. A generalized linear model was used to perform a multivariable analysis. All statistical analyses were performed using STATA/IC 13 for Mac (StataCorp LP, College Station, TX, USA) and significance assigned on the basis of 95% confidence intervals and p<0.05.

## Results

**Fig 1:**
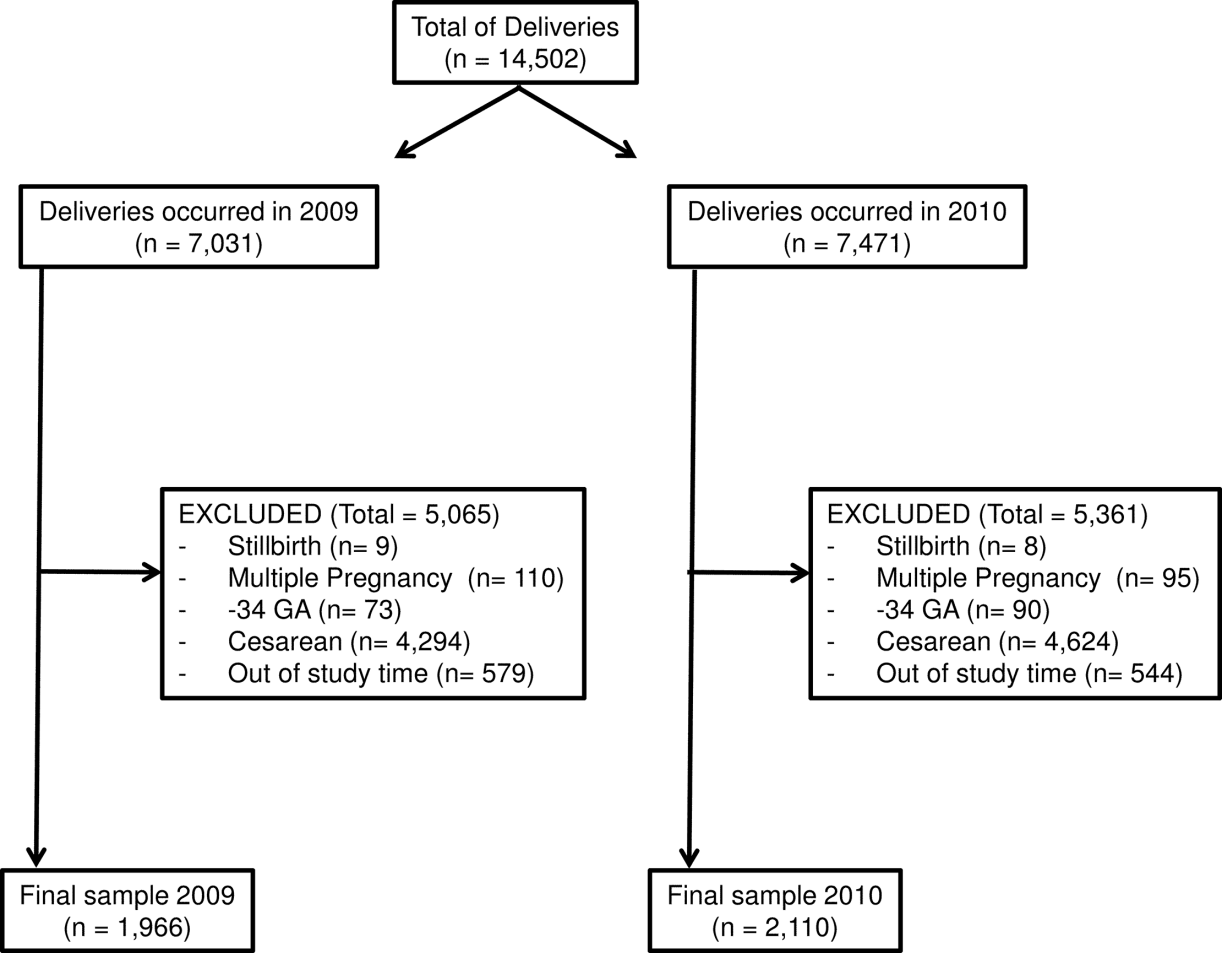
Flow diagram of selection of data.

A total of 1,966 deliveries from 2009 and 2,110 from 2010 were included in the final analysis (Fig 1). A similar distribution of males was observed between the two groups: 48.2% (948) boy in 2009 and 49.3% (1,041) in 2010 (p = 0.476).

Maternal age was statistically significantly different between the two cohorts. Mothers who delivered in 2010 were on average older than mothers who delivered in 2009 (p = 0.001). Parity ranged between 0 and 5, and most of the mothers were primigravidas with no significant difference between 2009 and 2010 (p = 0.07). Most of the subjects belong to the low annual income group, which was not significantly different between the groups (p = 0.06) (Table 1).

**Table 1:** Maternal and newborn characteristics and perinatal outcomes by year and gender.

Variable	2009		2010	
	Males (n = 948)	Females (n = 1,018)	Males (n = 1,041)	Females (n = 1,069)
Maternal Age, year^a^	27 (24–31)		28 (25–32)*	
Primigravida, n (%)	949 (52.84)		956 (48.85)	
Income, n (%)				
Low	1,123 (57.12)		1,157 (54.83)	
Middle	706 (35.91)		767 (36.35)	
High	137 (6.97)		186 (8.82)	
GA, weeks^a^	39 (38–40)		39 (38–40)	
	39 (38–40)	39 (38–40)	39 (38–40)	39 (38–40)
GA, days^a^	273 (266–280)		273 (266–280)	
	273 (266–280)	273 (266–280)	273 (266–280)	273 (266–280)
SGA, n (%)	134 (6.82)		173 (8.2)	
	61 (6.43)	73 (7.17)	75 (7.2)	98 (9.17)
Preterm, n (%)	55 (2.8)		60 (2.84)	
	30 (3.16)	25 (2.46)	35 (3.36)	25 (2.34)
Weight, grams^b^	3,358±377.19		3352.29±392	
	3,400.20±384.3	3,318.63±366.28	3,386±400.18	3,319.47±381.19
Length, cm^a^	50 (49–51)		49.5 (48.5–51)*	
	50 (49–51)	50 (48.5–50.5)	50 (48.5–51)**	49.5 (48–50.5)**
HC, cm^a^	35 (34–35.5)		34.5 (34–35.5)*	
	35 (34–36)	34.5 (34–35)	35 (34–35.5)**	34.5 (34–35)**
PI, gr/m^3^ ^a^	2.69 (2.56–2.82)		2.74 (2.59–2.9)*	
	2.67 (2.54–2.81)	2.7 (2.57–2.84)	2.74 (2.59–2.9)**	2.75 (2.6–2.91)**

^a^ Results as reported as median (percentile 25 –percentile 75)

^b^ Results as reported as mean ± standard deviation

* P ≤0.05: significant difference between years

** P ≤0.05: significant difference between sex and years

NE: Non-exposed; E: Exposed; GA: Gestational Age; SGA: Small Gestational Age; HC: Head Circumference; PI: Ponderal Index

### Characteristics of exposed versus non-exposed newborns by gender

Comparing the 2009 and 2010 cohorts, no statistically significant differences were identified between gestational age at birth (p = 0.2), the proportion of SGA (p = 0.232) or preterm deliveries (p = 0.929), and birth weight (p = 0.636). Babies delivered in 2010; however, were on average of shorter length (p<0.05), had a smaller head circumference (p = 0.001) and had a greater ponderal index (p = 0.001) than their counterparts born in 2009 (Table 1).

Analysis by year and gender identified no statistically significant difference in birth weight among males (p = 0.418) and females (p = 0.959), and no difference in gestational age at birth. The length and head circumference; however, were statistically significantly different (p<0.05), with exposed males and females being shorter and having a smaller head circumference than those in the non-exposed cohort (Table 1).

### Perinatal outcomes of exposed versus non-exposed newborns by gender and trimester of exposure

Birth weight (either in males or females) was not significantly affected by the trimester of exposure. Exposed male fetuses, however, displayed measurable, significant differences in length, head circumference, and gestational age at birth according to gestational age at the time of exposure. Table 2 presents perinatal outcomes of male newborns according to their gestational age at the time of the earthquake. For instance, males exposed in their second and third trimester were born smaller than non-exposed males (p = 0.001). Moreover, males exposed in their second trimester had smaller head circumference than non-exposed males, and their gestational age was shorter. Ponderal index was greater if male newborns were exposed during the second and third trimester.

**Table 2:** Perinatal outcomes of males according to trimester of exposure to earthquake.

Variable	1^st^ trimester			2^nd^ trimester			3^rd^ trimester		
	NE (n = 288)	E (n = 390)	p-value	NE (n = 319)	E (n = 329)	p-value	NE (n = 341)	E (n = 322)	p-value
GA, weeks^a^	39 (38–39)	39 (38–39)	0.397	39 (38–40)	39 (38–39)	0.004*	39 (38–39)	39 (38–40)	0.172
GA, days^a^	273 (266–273)	273 (266–273)	0.397	273 (266–280)	273 (266–273)	0.004*	273 (266–273)	273 (266–280)	0.172
Weight, gr^b^	3,400±398.85	3,374.07±397	0.663	3403.1±392.79	3375.82±403.5	0.383	3408.39±364.06	3410.84±400.8	0.934
Length, cm^a^	50 (49–51)	50 (49–51)	0.156	50 (49–51)	49.5 (48.5–51)	0.001*	50.5 (49–51)	50 (49–51)	0.001*
HC, cm^a^	35 (34–35.5)	35 (34–35.5)	0.204	35 (34–36)	35 (34–35.5)	0.002*	35 (34–36)	35 (34–35.5)	0.612
PI, gr/m^3^ ^a^	2.69 (2.55–2.82)	2.7 (2.57–2.87)	0.123	2.68 (2.53–2.8)	2.76 (2.63–2.9)	0.001*	2.66 (2.53–2.79)	2.75 (2.62–2.88)	0.001*

^a^ Results reported as median (percentile 25 –percentile 75)

^b^ Results reported as mean ± standard deviation

* P ≤0.05: significant difference between exposed and non-exposed male newborns

NE: Non-exposed; E: Exposed; GA: Gestational Age; SGA: Small Gestational Age; HC: Head Circumference; PI: Ponderal Index

In contrast, female fetuses exposed in the second and third trimester were significantly shorter than non-exposed females without a significant difference in birth weight. They had a smaller head circumference if exposure occurred during the second trimester. As such, their ponderal index was similarly affected. Table 3 presents perinatal outcomes of female newborns according to their gestational age at the time of the earthquake.

**Table 3:** Perinatal outcomes of females according to trimester of exposure to earthquake.

Variable	1^st^ trimester			2^nd^ trimester			3^rd^ trimester		
	NE (n = 325)	E (n = 368)	p-value	NE (n = 350)	E (n = 337)	p-value	NE (n = 343)	E (n = 364)	p-value
GA, weeks^a^	39 (38–40)	39 (38–40)	0.709	39 (38–40)	39 (38–39)	0.256	39 (38–40)	39 (38–40)	0.456
GA, days^a^	273 (266–280)	273 (266–280)	0.709	273 (266–280)	273 (266–273)	0.256	273 (266–280)	273 (266–280)	0.456
Weight, gr^b^	3333.89±376.86	3334.29±377.99	0.988	3305.74±359.87	3330.8±387.19	0.379	3317.32±363.13	3293.99±378.5	0.403
Length, cm^a^	49.5 (48.5–50.5)	49.5 (48–50.5)	0.474	50 (49–50.5)	49 (48–50.5)	0.001*	50 (48.5–51)	49 (48–50.5)	0.001*
HC, cm^a^	34.5 (33.5–35)	34.5 (34–35)	0.353	34.5 (34–35)	34.5 (33.5–35)	0.268	34.5 (34–35.3)	34.5 (34–35)	0.026*
PI, gr/m^3^ ^a^	2.73 (2.61–2.87)	2.74 (2.59–2.9)	0.459	2.68 (2.55–2.83)	2.77 (2.6–2.96)	0.001*	2.69 (2.56–2.83)	2.73 (2.58–2.9)	0.011*

^a^ Results reported as median (percentile 25 –percentile 75)

^b^ Results reported as mean ± standard deviation

* P ≤0.05: significant difference between exposed and non-exposed female newborns

NE: Non-exposed; E: Exposed; GA: Gestational Age; SGA: Small Gestational Age; HC: Head Circumference; PI: Ponderal Index

### Multivariable analysis

After adjusting for gender, income, gestational age (either in weeks or days), maternal age and parity, women exposed in their first trimester had newborns who were significantly shorter (by around 2 mm) compared to women who were not exposed (95% CI, 0.35–0.02; p = 0.023). Women exposed in their second trimester had newborns that were on average 5 mm shorter than their unexposed newborns (95% CI, 0.64–0.31; p = 0.001). Women exposed in their third trimester had newborns were on average 4.5 mm shorter than unexposed newborns (95% CI, 0.62–0.28; p = 0.001), holding all variables constant.

Birth weight was related to newborn gender, gestational age at birth (either in weeks or days), and parity, but was not significantly influenced by trimester of exposure. After adjusting for birth weight and length, the ponderal index was affected by exposure to earthquake in the second and third (but not the first) trimester. For example, for exposure in the second trimester, the ponderal index was greater by 0.09 in exposed compared with non-exposed fetuses (95% CI, 0.06–0.11; p = 0.001) and by 0.07 if exposure was in the third trimester (95% CI, 0.04–0.9; p = 0.001), regardless of gender.

Head circumference was associated with exposure if it occurred in the first and second trimester of gestation. Newborns exposed during their first trimester had a head circumference on average 1.2 mm smaller than unexposed newborns (95% CI, 0.23–0.003; p = 0.04) and, if exposure occurred during second trimester, the head circumference was on average 1.5 mm less (95% CI, 0.27–0.03). The Apgar score at 5 minutes was not affected by exposure.

## Discussion

The current study analyzes the effect of the historic February 27, 2010 earthquake on perinatal and newborn outcomes in a large tertiary care center in Santiago, Chile. We found relationships between this natural disaster and adverse perinatal outcomes that varied depending on the timing of exposure and newborn gender. We obtained significant findings on the effects of the earthquake on fetal length, head circumference, ponderal index, and gestational age at birth.

Prior studies have proposed a negative relationship between prenatal maternal stress and gestational length; that is, women under stress due to a natural disaster have a lower gestational age at delivery than women without stress [9,23–25]. We found a similar difference between exposed and non-exposed males; however, the earthquake did not appear to have the same effect on gestational age at delivery if the fetus was female. These data are consistent with the findings of Tan et al. [6]. The association between PNMS and an increased risk of preterm birth has been previously described [10,18,25,26], although the effect of timing of exposure and fetal gender had not been systematically examined. We found that male newborns had shorter gestation if they were exposed during their second trimester of gestation, in contrast to female newborns, where no such association was evident, suggesting that the risk of shorter pregnancy varied according to fetal gender. Interestingly, Torche et al. found a greater probability of preterm birth among females than among males after exposure to a natural disaster [25].

Although the published data about the effect of natural disasters on the head circumference are discrepant, most authors observed a smaller head circumference [10,27]; Tegethoff et al., however, found otherwise [24]. We observed that head circumference was smaller for male newborns, especially if they were exposed during the second trimester of gestation, whereas female newborns had smaller head circumference if they were exposed in their third trimester. Both male and female neonates were shorter if they were exposed during their second and third trimester. Most prior studies did not show a difference in fetal length following a disaster, except for Dancause et al. who observed smaller length of newborns among women exposed to a catastrophic ice storm in Canada [28].

Several prior studies have suggested that PNMS is associated with low birth weight [2,5,6,12]. A special case is presented by Harville et al. who observed that newborns exposed to 2010 earthquake in Haiti had on average 150 to 300 grams less that non-exposed [29]. In this study, we did not find a significant difference in birth weight between groups. Although, because the length was shorter in exposed newborns, the ponderal index was significantly greater in exposed than non-exposed newborns. In contrast, Oyarzo et al. reported that exposed newborns had a greater length and thus a lower ponderal index. A possible explanation for this difference is that Oyarzo et al. observed pregnant women exposed much closer to the epicenter of earthquake and it is possible that the intensity of the stress injury could affect growth differently [3]. Consistent with this hypothesis, Suzuki et al. reported that newborn birth weight was extremely affected if pregnant women were close to the most affected area compared to other geographical locations [12].

Some of these findings can be explained because maternal stress and perinatal outcomes are linked both psychologically and endocrinologically [9]. PNMS can affect the fetal hypothalamic pituitary adrenal (HPA) axis [30] and its downstream metabolic pathways [31]. The effect of PNMS appears to be mediated through altered production of corticotrophin-releasing hormone (CRH), adrenocorticotrophic hormone (ACTH), and cortisol [9]. These stress hormones may increase uterine contractions and the timing of parturition, thus changing the length of gestation [32,33]. They may also affect placental function [34], which is an important maternal-fetal barrier, leading to fetal growth restriction, prematurity, and low birth weight [35–38]. Prenatal maternal stress and high levels of maternal cortisol have been reported as factors that can induce epigenetic modifications that may affect health outcomes in later life [39], also they can modify gene expression in fetal brain [40], and maybe these changes are fetal sex-specific in their manifestation. Additionally, Wang et al. reported that women exposed to prenatal stress due to an earthquake had short leukocyte telomere length, what has been associated with an increased disease risk in adulthood [41].

This study has important implications for future research and medical practice. First, as far as public policy is concerned, it would be important to develop and implement a specific program to support pregnant women and help them cope with the stress of a natural disaster. Second, women who have been exposed to acute stress due to a natural disaster should be followed more closely during their prenatal course to look for symptoms and signs of preterm labor, especially if the fetus is male. Finally, further research should focus on investigating the long-term outcomes related to different natural disasters and providing support to children who experience sequelae from a stressful event experienced in utero. It is well known that such negative experienced during the fetal and early neonatal period can lead to an increase in years of life lost (YLL), disability-adjusted life years (DALYs), and quality-adjusted life years (QUALYs) due to metabolic and endocrine diseases, as well as psychological and neurological alterations including emotional, cognitive, and linguistic impairments. This assumption is supported by one study that evaluated the acute impact on brain function in people who survived to an 8.0 earthquake in China, through magnetic resonance imaging. As a result, victims presented several alterations across the brain, such as hyperactivity and low functional connectivity [42]. Furthermore, all of maternal physiological alterations can affect to fetus. One study found that newborns exposed to prenatal stress due to an earthquake presented an inverse relationship between the level of maternal stress and scores of mental development, without differences among trimesters. Specifically, males were more affected by this stress in comparison to females [43].

This study has several strengths. First, the data were abstracted from a research quality medical database without missing data points. Second, the data were collected from subjects that had attended the medical center over a two-year period; as a result, the demographic characteristics between exposed and non-exposed women were similar. Third, the medical center under study represents almost 7% of all deliveries occurred in Chile in that date [44].

One limitation of this study is the inability to include some variables of interest, such as the underlying details of maternal chronic diseases or the length of prior pregnancies. This center has a high cesarean delivery rate (around 50%), some of which are conducted electively on maternal request. Unfortunately, the database does not distinguish between elective and non-elective C-sections. Consequently, in order to avoid bias, we limited this analysis only to those women who delivered vaginally. While this helped remove many potential confounding factors from the analysis, it also leads to a decrease in the number of cases overall. Moreover, dataset contained gestational age at birth as entire weeks; so, a detailed gestational age of days would deliver a more accurate analysis. Even though the differences in fetal length and head circumference are statistically different, the absolute measurement is relatively small, and it may impress unimportant; however, these few millimeters can hide several molecular and physiology changes that will have several consequences as we exposed previously.

In conclusion, this study reinforces the hypothesis that natural disasters are associated with adverse perinatal outcomes and that a single severe acute maternal stress event may affect fetal growth and timing of delivery. This relationship, in turn, appears to be dependent on fetal gender and on the gestational age at which the stress event occurred. Further research, a cellular and molecular level, is needed to better understand the relationship between maternal stress events, and perinatal and childhood outcomes.
